# Basic Color Terms (BCTs) and Categories (BCCs) in Three Dialects of the Spanish Language: Interaction Between Cultural and Universal Factors

**DOI:** 10.3389/fpsyg.2018.00761

**Published:** 2018-05-18

**Authors:** Julio Lillo, Fernando González-Perilli, Lilia Prado-León, Anna Melnikova, Leticia Álvaro, José A. Collado, Humberto Moreira

**Affiliations:** ^1^Departamento de Psicología Social, del Trabajo y Diferencial, Facultad de Psicología, Universidad Complutense de Madrid, Madrid, Spain; ^2^Facultad de Información y Comunicación, Instituto de Comunicación, Universidad de la República, Montevideo, Uruguay; ^3^Centro Universitario de Arte, Arquitectura y Diseño, Universidad de Guadalajara, Jalisco, Mexico; ^4^Anglia Vision Research, Department of Vision and Hearing Sciences, Anglia Ruskin University, Cambridge, United Kingdom; ^5^The Sussex Colour Group, School of Psychology, University of Sussex, Falmer, United Kingdom

**Keywords:** basic color terms, basic color categories, universal factors, linguistic relativism, Spanish, Castilian, Mexican, Uruguayan

## Abstract

Two experiments were performed to identify and compare the Basic Color Terms (BCTs) and the Basic Color Categories (BCCs) included in three dialects (Castilian, Mexican, and Uruguayan) of the Spanish language. Monolexemic Elicited lists were used in the first experiment to identify the BCTs of each dialect. Eleven BCTs appeared for the Spanish and the Mexican, and twelve did so for the Uruguayan. The six primary BCTs (*rojo* “red,” *verde* “green,” *amarillo* “yellow,” *azul* “blue,” *negro* “black,” and *blanco* “white”) appeared in the three dialects. This occurred for only three derived BCTs (*gris* “gray,” *naranja* “orange,” and *rosa* “pink”) but not for the other five derived BCTs (*celeste* “sky blue,” *marrón* “brown,” *café* “brown,” *morado* “purple,” and *violeta* “purple”). Color transitions were used in the second experiment for two different tasks. Extremes naming task was used to determine the relation between two different dialects' BCTs: equality, equivalence or difference. The results provided the first evidence for *marrón* “brown” and *café* “brown” being equivalent terms for the same BCC (brown in English) as is the case of *morado* “purple” and *violeta* “purple.” Uruguayan *celeste* “sky blue” had no equivalent BCT in the other two dialects. Boundary delimitation task required the selection of the color in the boundary between two categories. The task was used to reasonably estimate the volume occupied by each BCC in the color space considering its chromatic area and lightness range. Excluding sky blue (*celeste* “sky blue”) and blue (*azul* “blue”), the other BCCs color volumes were similar across the three dialects. Uruguayan sky blue and blue volumes conjointly occupied the portion of the color space corresponding to the Castilian and Mexican blue BCC. The fact that the BCT *celeste* “sky blue” only appeared in Uruguayan very probably derived from specific cultural factors (the use of the color in the flags and the arrival of an important number of Italian immigrants). Nevertheless, these cultural factors seem to nurture from a perceptive structuring of the color space, which nature is universal, as the boundaries of this category can be delimited from the responses of Spanish and Mexican participants.

## Introduction

Normal trichromats are able to differentiate more than 2 million colors (Pointer and Attridge, 1998; Kuehni, 2016) which can be clustered in a greatly reduced number of color categories. This reduction happens because thousands of colors differing in lightness and/or chroma and/or hue can belong to a single category and, consequently, be denoted by the same term. Such term is considered a Basic Color Term (BCT) that identifies a Basic Color Category (BCC) when it is used consistently among most speakers of a language (Berlin and Kay, 1969; Crawford, 1982; Corbett and Davies, 1997; Hardin and Maffi, 1997).

In our current work, we identified the BCTs of three Spanish dialects—Castilian, Uruguayan, and Mexican—and delimited the space of their corresponding BCCs. This identification and delimitation enabled us to, first, detect any possible significant change in number and characteristics of the Castilian BCTs-BCCs regarding our previous work (Lillo et al., 2007), second, to compare BCCs-BCTs between the aforementioned dialects, and, third, to interpret our data in reference to the Linguistic Relativity Hypothesis (LRH) (Saunders and van Brakel, 1997; Roberson et al., 2000; Davidoff, 2015) and the model of Universals and Evolution (UE) (Berlin and Kay, 1969; Kay and Maffi, 1999; Kay et al., 2009).

In our previous work (Lillo et al., 2007), Elicited lists indicated that there were 11 BCTs in Castilian (see also Uusküla and Bimler, 2016). Moreover, several naming tasks allowed us to map the volumes of the Castilian BCCs and to conclude that their colorimetric delimitation was similar to that of American English (Boynton and Olson, 1987; Lindsey and Brown, 2014), British English (Sturges and Whitfield, 1995), Chinese (Lin et al., 2001), and Japanese (Uchikawa and Boynton, 1987) BCCs. As in Castilian, there were 11 BCCs-BCTs in these languages.

At the time our previous work was published (Lillo et al., 2007), 11 BCCs-BCTs were reported for Castilian and Japanese. This means that these languages were then considered to divide color space into 11 parts (volumes). However, in a recent work, Kuriki et al. (2017) demonstrated development of a 12th BCT-BCC in Japanese. As in the case of Russian (Paramei, 2007), Greek (Androulaki et al., 2006), and Italian (Paggetti et al., 2016), the 12th Japanese category (labeled as *mizu* “sky blue”) evolved from partition of an originally more extended color volume of the previous Japanese blue (labeled *ao* “blue,” Uchikawa and Boynton, 1987), similar to the English blue, into two more constrained category-volumes. One of the new categories corresponds to darker blues, and the other one encompasses light blues and is labeled *mizu* “sky blue.” Considering this, one of our aims was to find out whether a similar process could have taken place in Castilian, as well as to find out whether a similar 12th category already exists in Uruguayan, as in Guatemalan Spanish (Harkness, 1973, Figure 9), which previous work seems to indicate (González-Perilli et al., 2017).

There are important differences between languages in the number and colorimetric extension of their BCTs-BCCs. Languages spoken in pre-technological cultures (MacLaury, 1997; Kay et al., 2009) tend to have a reduced number of BCCs. Fewer categories imply that either more colors must be named with the same BCT or some parts of the color space are not named consistently. At this point a question about how new BCCs appear may be posed, so let us take a closer look at two major theoretical approaches; Linguistic Relativity Hypothesis (LRH), and Universals and Evolution Hypothesis (UEH).

According to LRH (Saunders and van Brakel, 1997; Davidoff et al., 1999; Roberson et al., 2000; Davidoff, 2015), categorical segmentation of color space arises from the socio-cultural need to discuss meaningful properties of object surfaces. Consequently, in the first stage of the evolution of BCTs, some terms could only be used for the naming of some important items, and, considering the partial nature of color constancy (McCann et al., 2014; Allred and Olkkonen, 2015), these terms could be related to the small subset of color experiences produced by such items in different illumination backgrounds. Subsequently, the similarity between such colors and those produced by other items could promote the acquisition of the generic use characteristic of the BCTs in the developed languages. This interpretation can explain that, for example, the word “red” in the Indo-European languages seems to derive from a word for blood; however, the term “red” now denotes a group of similar colors and not a specific object (Heller, 2009; Biggam, 2010).

LRH assumes only one universal limitation related to the origin and evolution of BCCs: color proximity. That is, the colors included in a BCC must be contiguous in color space. Besides this requisite, LRH considers color space as a *tabula rasa* where linguistic-cultural factors operate freely. On the contrary, according to UEH the color space is not a *tabula rasa* because BCCs' origin and evolution also depend on other universal factors related to color perception.

UE model postulates (Kay et al., 1991, 2009; Kay and Maffi, 1999; Kay, 2015) that the sensations related to the 6 Hering primary colors (red, green, yellow, blue, white, and black) are universals that are not dependent on the exposure to concrete experiences and partially determine BCCs' origin and evolution in any language. In line with this idea, some works have shown that Hering chromatic opponent hue sensations (red-green; yellow-blue) can result from the combination of the S, M, and L cone responses in some neurons of the outer retina (Schmidt et al., 2014). The sign of the L-M balance seems to determine which color sensations can (or cannot) coexist in compound BCCs (Xiao et al., 2011). Event Related Potentials (ERP) in pre-linguistic 7-month old infants (Clifford et al., 2009) support categorical responses to color (along the green-blue variation) before color terms are acquired. Also using ERPs, a recent study (Forder et al., 2017) found that ERP component P2 differentiates between unique hues (i.e., only associated with one of Hering's chromatic sensations) and binary hues (i.e., associated with two of Hering's chromatic sensations). Considering all of the above, if it is assumed that chromatic sensations derive from the activity of universal perceptual mechanisms, and if it is also assumed, as in the UEH, that the chromatic categories are based in such sensations, then similarities are to be expected between languages.

LRH and UEH differ in how they explain the appearance of new BCTs. LRH endorses the Emergence Hypothesis (Levinson, 2000), according to which languages only develop BCTs/BCCs for areas of color space which require consistent naming. In languages of technologically developed societies such a requirement would appear for the naming of colors at the boundaries of initial BCCs. For example, the emergence of a novel sky blue category would result from the need to communicate efficiently, and to name consistently, the colors at the boundary between the blue and white categories. On the other hand, the UEH explains the apparition of new BCCs as an outcome of successive differentiation. In this way a novel sky blue would emerge as the result of partitioning the volume of the color space previously named by only one BCT (*azul* “blue”). Lindsey and Brown's (2014) recent work on American English color categories shows that successive differentiation and emergence processes can coexist. Some American color terms that could be in the throes of becoming BCTs refer to colors in the boundary between 2 established BCCs-BCTs (e.g., turquoise, teal, and aquamarine are in the boundary between green and blue), as the Emergence Hypothesis requires. At the same time, other color terms (e.g., lavender and lime) begin to be used consistently for naming some colors included into established BCCs-BCTs (purple and green, respectively), as required by the successive differentiation mechanism.

To make the comparison between our previous work (Lillo et al., 2007) on Castilian BCCs-BCTs and the current work (Castilian related to other two Spanish dialects, Uruguayan and Mexican) easier, we used the same task (Elicited lists) and types of analysis (frequency and the position of the terms in the lists). Considering that in the UEH the primary categories (red, green, blue, yellow) have a special status because of being directly associated to 1 of 6 Hering's elemental sensations, we predict that these primary categories would appear earlier and more frequently (as it was observed in Lillo et al., 2007), and would present a higher consistence between the three dialects (i.e., the same terms would be observed in all three dialects).

In our previous work (op. cit.), BCC colorimetric mapping was carried out using the results of several naming tasks, in which all the stimuli (more than 1,500) of the NCS (Natural Color system) color atlas were used. Such a high number of stimuli imply long and uninteresting experimental sessions for the participants. To avoid this inconvenience, we used representative color samples with fewer stimuli, as had been done in previous work on the use of BCTs by color deficient people (Lillo et al., 2012a,b, 2014; Moreira et al., 2014) or on their color preferences (Álvaro et al., 2015).

**Figure 1:**
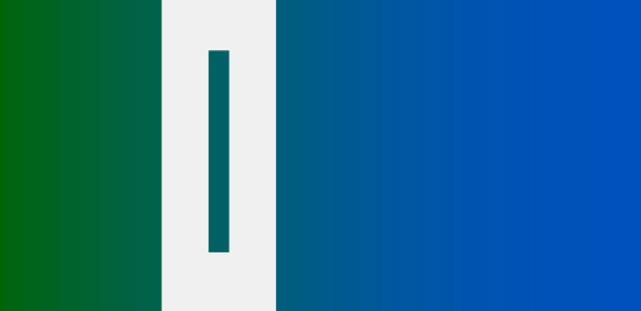
Example of color transition. Each color presented in an extreme was a good exemplar of a BCC. Task 1 (extremes naming) required the naming of the color in each transition extreme. Task 2 (boundary delimitation) required to place the whitish gray rectangle gap on the color halfway between the two categories represented in the extremes of each transition.

On the other hand, a reduced sample makes it difficult to perform precise BCCs' colorimetric mappings. That is, a reduced number of stimuli makes it difficult to find colors in the boundaries between two categories. To overcome this limitation, and to avoid big stimulus samples, we used color transitions in our current research (see Figure 1) to perform two different tasks. First the extremes naming task required the naming of the colors of the extremes of each transition. This task was used to specify the type of relation between two BCTs belonging to two different dialects. Relation type 1 was BCT equality: The same BCT (e.g., *rojo* “red”) was used for the naming of the same colors in two dialects. Relation type 2 was BCT equivalence: colors named A in dialect X were named B in dialect Y. That is, although a BCT may differ from one dialect to another, it named the same color set (e.g., *marrón* “brown” in Spanish and *café* “brown” in Mexican). Finally, relation type 3 was difference: confirmed by colors with different BCTs with neither equality nor equivalence relation.

The second color transitions task was named “boundary delimitation task,” and was used to make a reasonable estimation of the volume occupied by each BCC in the color space. The boundary delimitation task was performed after the extremes naming task. It required the selection of the color categorically halfway between the two categories appearing in the extremes of the transition, i.e., the color equally likely to be labeled with either term. We assumed that the colors between an extreme and a boundary belonged to the part of the color space related to the BCT used for naming the extreme. For example, the term *rojo* “red” can be used for naming all the colors between the good exemplar of red category in the extreme and the boundary with another category (i.e., orange or brown). We expected similar color volumes for the BCTs pairs with type 1 (equality) and type 2 (equivalence) relations, but different color volumes for the type 3 relations.

The design of our boundary delimitation task shows some similarities to that employed for the same purpose, in Castilian Spanish, by Parraga and Akbarinia (2016; see also Párraga et al., 2009). However, some important differences need to be pointed out. First, Parraga and Akbarinia (2016) presented color singletons, not color transitions, which may have increased the difficulty of their task. Second, the participants' task was to adjust the stimulus color until they found it to be on the boundary between the two categories. Third, prior to each trial, participants were provided with a message (on a monitor) that explicitly named the two categories in question. The extremes naming task in the present study deliberately avoided imposing color names onto participants in this way, with the aim of finding out the equivalence between BCTs used for naming the BCCs in each dialect group.

To facilitate the comparison of our current data to the previous data on Castilian BCTs-BCCs colorimetric delimitation (Lillo et al., 2007), a strict differentiation between qualitative (described by CIE *u*′, *v*′ chromaticity diagrams) and quantitative (described by CIE L^*^ variable) aspects was performed. Differences between BCCs colorimetric delimitations would indicate the influence of dialect-specific factors relating to the LRH postulates. Complementary analysis using other CIE variables will appear in book currently in preparation (Lillo et al., 2018).

In our previous work (Lillo et al., 2007) we did not find significant differences between males and females, neither in number of terms per list, or in the colorimetric delimitation of BCCs. Nevertheless, in our current research, we performed between-gender comparison, as some works relating to color denomination did find such differences (i.e., MacDonald and Mylonas, 2014).

## Materials and methods

### Experiment 1: elicited lists

#### Participants

Two hundred and one people (135 females, 66 males) from three different countries participated in the Elicited lists task. They all were university students. Table 1 shows their age distribution by country and sex. The research was conducted according to the principles expressed in the Declaration of Helsinki and all participants gave written informed consent. In all three universities, the research was approved by the relevant ethics committee: Hospital Clínico San Carlos Review Board in the Universidad Complutense de Madrid (Spain); Research Ethics Committee of the Faculty of Psychology in the Universidad de la República (Montevideo Uruguay); Scientific Committee of the Center of Art, Architecture and Design in the Universidad de Guadalajara (Mexico).

**Table 1:** Details of the participants in Experiment 1.

**Dialect**	**Females**	**Males**	**Total**
Castilian (UCM, Madrid, Spain)	35 (21.00; 2.30)	12 (21.42; 2.81)	47 (21.11; 2.46)
Mexican (U. Guadalajara; Mexico)	57 (18.91; 1.81)	40 (20.18; 2.22)	97 (19.43; 1.98)
Uruguayan (U. de la República; Montevideo, Uruguay)	43 (19.40; 1.87)	14 (19.50; 1.83)	57 (19.42; 1.85)
Total	135 (19.61; 2.01)	66 (20.25; 2.42)	201 (19.82; 2.17)

*The rows describe the three participant samples: Castilian, Mexican or Uruguayan (university of participants in brackets). The columns show the total number of participants, (mean and standard deviation of age in years in brackets)*.

#### Materials and procedure

Participants, organized in groups of 3–4 members, provided some identification data first and then carried out in silence a 2-min task that consisted of writing down on an individual piece of paper all the one-word color names they could recall. To avoid the influence of visual stimulation the whole procedure was performed with their eyes closed. We recorded the names and their order. Afterwards, the concise (14-plate) edition of the Ishihara pseudoisochromatic test (2006) was used to exclude those participants with red-green color deficient vision.

#### Results

We computed three variables from the individual lists data: (1) the number of terms in each list, (2) terms frequencies (number of lists, and their corresponding proportion, where each term appeared), and (3) relative order.

##### Number of terms

Considering the global group and each dialect group separately, four Man-Whitney *U*-tests showed no significant differences between females and males. The means, U and probability values were: Global, males = 12.84, females = 12.54, *U* = 3704, *p* = 0.563. Castilian, males = 10.41, females = 10.91, *U* = 176.50, *p* = 0.41. Mexican, males = 13.72, females = 12.96, *U* = 960.5, *p* = 0.185. Uruguayan, males = 12.43, females = 13.32, *U* = 237, *p* = 0.231.

A non-parametric variance analysis indicated that there were significant differences (χ^2^ = 27.165, *p* < 0.001) in the number of terms between the three dialects. The application of a series of Man-Whitney *U*-tests revealed that the number of terms in the Castilian dialect (10.78) was smaller than in the Mexican (13.27; *U* = 1071.5, *p* < 0.01) and the Uruguayan (13.10; *U* = 957, *p* < 0.01). There were no significant differences in the number of terms between Mexican and Uruguayan dialects (*U* = *3292, p* = 0.522).

##### Frequency

**Figure 2:**
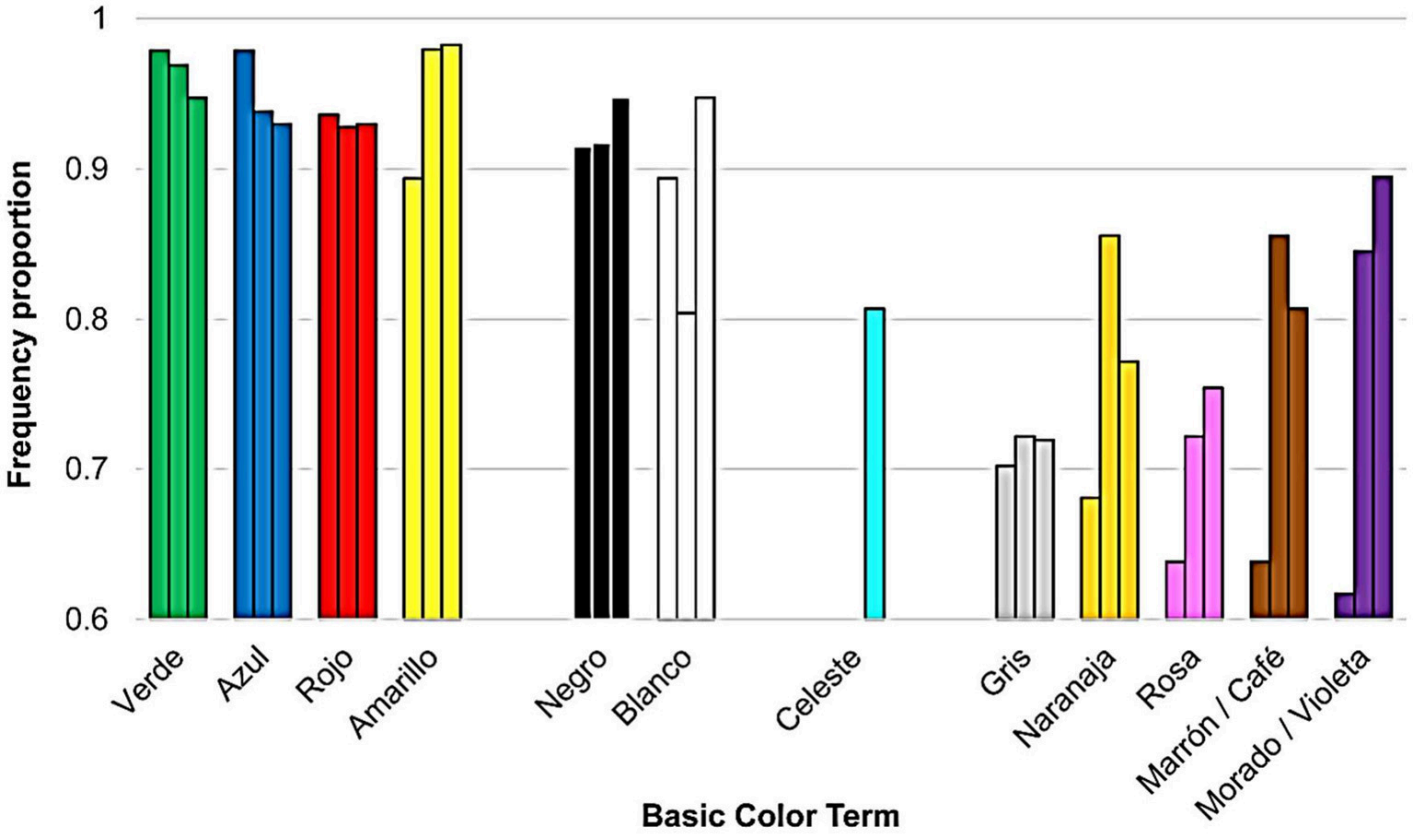
Elicited lists BCTs' frequency proportion (*y*-axis) for each basic color term (*x*-axis) from Experiment 1, in the three dialects of Spanish (Castilian, left; Mexican, center; Uruguayan, right). Note that *celeste* “blue sky” is only present in Uruguayan.

Table 2 and Figure 2 show the frequency results. Table 2 shows the frequency and the percentage for the terms appearing in more than 50% of the lists for each dialect. Table 2 also shows two terms nearest to this requisite for each dialect. The first column of Table 2 provides also English equivalents to Spanish terms. Most of these equivalences are based upon Lillo et al. (2007).

**Table 2:** The frequencies and percentages of the terms reported on Elicited lists in Experiment 1.

**Terms**	**Castilian (%)**	**Mexican (%)**	**Uruguayan (%)**
*Verde* “Green”	46 (98.79)	94 (96.90)	54 (94.74)
*Azul* “Blue”	46 (97.87)	91 (93.81)	53 (92.98)
*Rojo* “Red”	44 (93.62)	90 (92.78)	53 (92.98)
*Amarillo* “Yellow”	42 (89.36)	95 (97.94)	56 (98.25)
*Negro* “Black”	43 (91.49)	89 (91.75)	54 (94.74)
*Blanco* “White”	42 (89.36)	78 (80.41)	54 (94.74)
*Celeste* “Sky blue”	01 (00.02)	09 (00.09)	46 (80.70)
*Gris* “Gray”	33 (70.21)	70 (72.16)	41 (71.93)
*Naranja* “Orange”	32 (68.09)	83 (85.57)	44 (77.19)
*Rosa* “Pink”	30 (63.83)	70 (72.17)	43 (75.44)
*Marrón* “Brown”	30 (63.83)	00 (00.00)	46 (80.70)
*Café* “Brown”	00 (00.00)	83 (85.57)	00 (00.00)
*Morado* “Purple”	29 (61.70)	82 (84.54)	02 (03.51)
*Violeta* “Purple”	19 (40.43)	38 (39.18)	50 (89.47)
*Beige* “Beige”	10 (21.28)	47 (48.45)	10 (17.54)
*Fucsia* “Fuchsia”	04 (08.51)	23 (23.71)	22 (38.60)
*Lila* “Lilac”	06 (12.77)	23 (23.71)	17 (29.82)

*The first column contains Spanish color terms (in italics) and their English equivalents (after the hyphen). The other columns indicate the frequencies (percentages in brackets) of all the terms fulfilling the basicness criterion (appearance in more than 50% of the lists of one or more dialects) for each dialect (Castilian, Mexican, and Uruguayan)*.

Nine BCTs appeared in the three dialects: six primary BCTs (Lillo et al., 2007; *verde* “green,” *azul* “blue,” *rojo* “red,” *amarillo* “yellow,” *negro* “black,” *blanco* “white”) and three derived BCTs (*gris* “gray,” *naranja* “orange,” and *rosa* “pink”). Two other derived BCTs appeared in two out of three dialects: *marrón* “brown” (Castilian and Uruguayan) and *morado* “purple” (Castilian and Mexican). Three BCTs appeared in only one dialect: *celeste* “sky blue” (Uruguayan), *violeta* “purple” (Uruguayan) and *café* “brown” (Mexican). The most used terms without matching the 50% criteria (non-BCTs) were *violeta* “purple” (for the Castilian and the Mexican), *beige* “beige,” *fucsia* “fuchsia” and *lila* “lilac.”

To facilitate between dialects comparisons, Figure 2 shows bars chunked into groups of three. Each group provides information about the ratio of the lists containing a given BCT in the Castilian (represented by the leftmost bar of each group), the Mexican (central bar) and the Uruguayan (rightmost bar) dialects. A group of 3 bars for each BCT's frequency proportion (left = Castilian; Central = Mexican; Right = Uruguayan). The sole exception was *celeste* “sky blue” represented by a single bar, as it was a BCT only for the Uruguayan. The data on BCTs equivalences enabled us grouping together *marrón* “brown” (Castilian and Uruguayan) and c*afé* “brown” (Mexican) in one group, and *morado* “purple” (Castilian and Mexican) and *violeta* “purple” (Uruguayan) in another group.

Several non-parametric statistical analyses were performed using χ^2^ to compare frequencies, Wilcoxon tests to compare the relative frequency of primary vs. derived BCTs, and Mann-Whitney *U*-tests to compare females vs. males. First, we conducted a comparison between the dialects of BCTs with either type 1 or type 2 relation (respectively, equality and equivalence; see the naming task results of the experiment 2). Second, we conducted within-dialect comparisons of BCTs frequencies.

There were significant between dialect differences in the frequency of only five BCTs: *amarillo* “yellow” (χ^2^ = 4.89, *p* < 0.05); *blanco* “white” (χ^2^ = 5.41, *p* < 0.05); *naranja* “orange” (χ^2^ = 4.89, *p* < 0.05); *marrón/café* “brown” (χ^2^ = 4.89, *p* < 0.05); *morado/violeta* “purple” (χ^2^ = 4.89, *p* < 0.05). Pairwise comparisons specified these differences as follows: *Amarillo* “yellow” and *morado/violeta* “purple” were less frequent in the Castilian sample than in either the Mexican sample (*amarillo* “yellow,” χ^2^ = 4.89, *p* < 0.05; *morado/violeta* “purple,” χ^2^ = 9.35, *p* < 0.01) or the Uruguayan sample (*amarillo* “yellow,” χ^2^ = 6.82, *p* < 0.01; *morado/violeta* “purple,” χ^2^ = 9.55, *p* < 0.01). *Naranja* “orange” and *marrón/café* “brown” were less frequent in the Castilian sample than in the Mexican sample (*naranja* “orange,” χ^2^ = 6.02, *p* < 0.05; *marrón/café* “brown,” χ^2^ = 8.86, *p* < 0.01).

To test whether primary BCTs appeared more frequently than derived BCTs, we computed the relative frequency of primary and derived BCTs for each participant (i.e., the proportion of BCTs from the total of primary or derived BCTs used by each participant). Considering the global group and each dialect group separately, four Wilcoxon tests showed significant differences between primary and derived BCTs. The means, *Z* and probability values were: Global, primary = 0.93, derived = 0.77, *Z* = −8.74, *p* < 0.001. Castilian, primary = 0.94, derived = 0.66, *Z* = −5.49, *p* < 0.001. Mexican, primary = 0.92, derived = 0.82, *Z* = −4.79, *p* < 0.001. Uruguayan, primary = 0.95, derived = 0.79, *Z* = −4.81, *p* < 0.001.

To compare the relative frequency of primary and the relative frequency of derived BCTs between females and males, Mann-Whitney *U* tests were performed for the global group and each dialect group separately. Only one comparison was statistically significant: Castilian females used derived BCTs significantly more than Castilian males (0.72 vs. 0.47, *U* = 90, *p* < 0.01).

The results of pairwise comparisons between the frequency of terms for Castilian, Mexican and Uruguayan samples are detailed below.

Within-dialect comparison on the frequency of Castilian BCTs showed that there were no significant differences in pairwise comparisons of primary terms (e.g., between *verde* “green” and *blanco* “white,” χ^2^ = 2.848, *p* = 0.203) or pairwise comparisons of derived terms (e.g., *gris* “gray” vs. *morado* “purple,” χ^2^ = 0.758, *p* = 0.514). Every primary term was used significantly (*p* < 0.05) more frequently than any derived term (e.g., *blanco* “white” vs. *gris* “gray” χ^2^ = 5.343, *p* < 0.05) and every basic term was used significantly more than any non-basic term (*p* < 0.05).

Within-dialect comparison on the frequency of Mexican BCTs revealed that no significant differences were found in any pairwise comparison for five out of six primary terms (e.g., *amarillo* “yellow” vs. *negro* “black,” χ^2^ = 0.264, *p* = 0.607) or any pairwise comparison between derived terms (excepting *naranja* “orange” vs. *gris* “gray,” χ^2^ = 5.227, *p* = 0.034). *Blanco* “white” was an exception as its frequency was significantly lower than that of any other primary term (all *p* < 0.05) and similar to that of any derived term (*p* > 0.05). There was a significant difference (*p* < 0.05) between the frequency of primary terms (more frequent) and derived terms (less frequent), apart from *azul* “blue” and *rojo* “red” (the least frequent term within primaries, see Table 2) which were not significantly different in frequency from *naranja* “orange,” *café* “brown,” and *morado* “purple” (the most frequent derived terms, see Table 2; *p* > 0.05). Every basic term was used significantly more than any non-basic term (*p* < 0.05).

Within-dialect comparisons of the frequency of Uruguayan BCTs revealed no significant differences in any pairwise comparison between the primary terms (f.e. *amarillo* “yellow” and *rojo* “red,” χ^2^ = 1.883, *p* = 0.364) or any pairwise comparison between the derived terms, with the exception of *violeta* “purple” vs. *gris* “gray” (χ^2^ = 5.632, *p* < 0.05) and *violeta* “purple” vs. *rosa* “pink” (χ^2^ = 3.88, *p* < 0.05). The most frequent primary terms (*amarillo* “yellow,” *blanco* “white,” *negro* “black,” and *verde* “green,” see Table 2) were used more frequently than the least frequent derived terms (*naranja* “orange,” *rosa* “pink” and *gris* “gray”; i.e., *negro* “black” and *naranja* “orange,” χ^2^ = 7.270, *p* < 0.05). Every basic term was used significantly more than any non-basic term (*p* < 0.05). The frequency of *celeste* “sky blue” was significantly lower than that of *amarillo* “yellow,” *blanco* “white” *negro* “black” and *verde* “green” (the most frequent primaries; *p* < 0.05) and similar to the frequencies of the remaining BCTs.

##### Order

**Figure 3:**
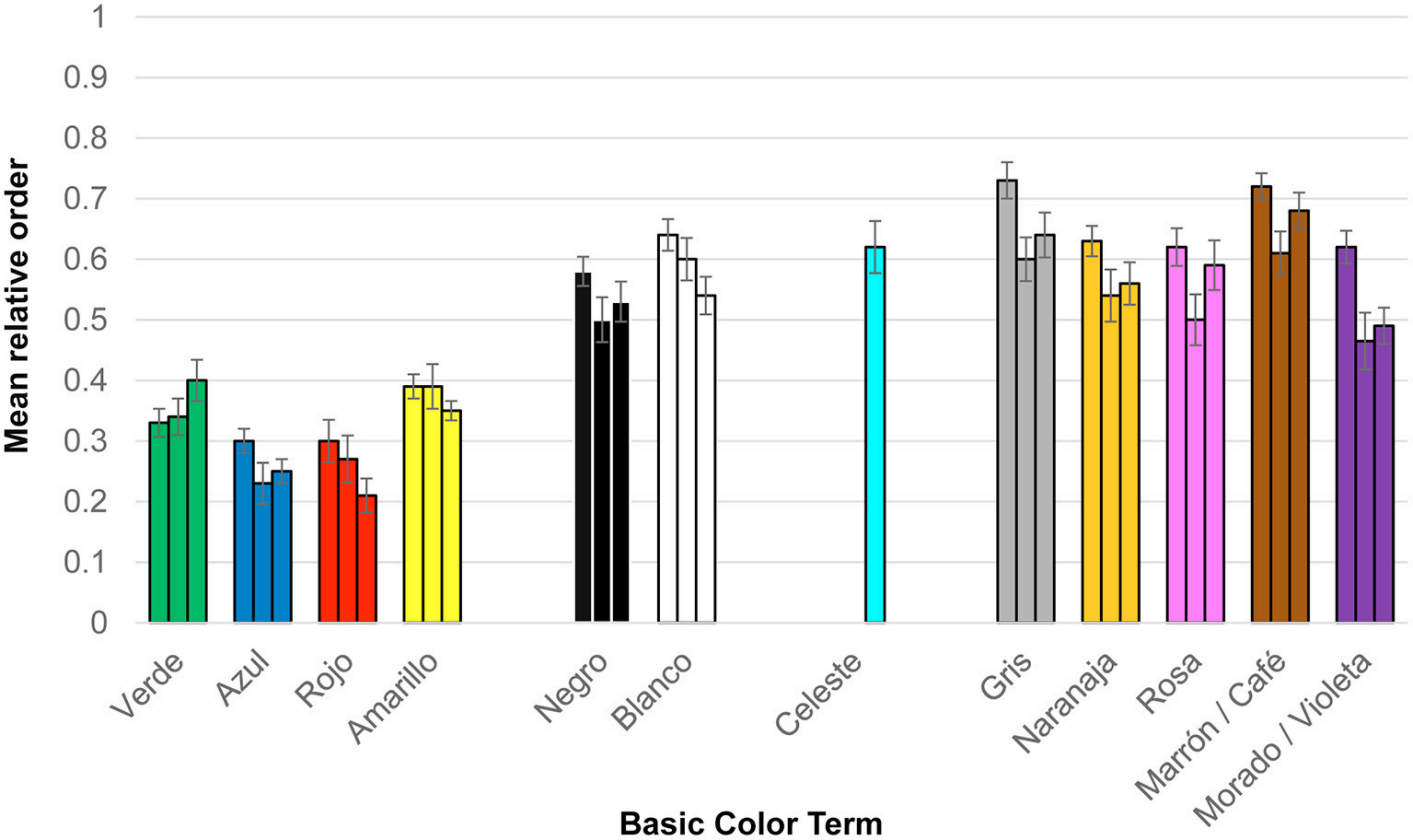
The mean relative order (*y*-axis) of BCTs in the Elicited lists task (Experiment 1), for each basic color term (*x*-axis) in the three dialects of Spanish (Castilian, left; Mexican, center; Uruguayan, right). Note that *celeste* “blue sky” is only present in Uruguayan.

To compensate for the differences in number of terms per list among the three dialects, and to facilitate the comparison between our current data and Lillo et al's. (2007) (Figure 1) data, we computed a relative order (O_R_) value for each color term in every individual list. We divided the absolute order (O_a_) by the number of terms (N) included in the target list (O_R_ = O_a_/N). Low O_R_ values indicate terms appearing at the beginning of the lists. Figure 3 shows mean relative orders of the BCTs in the three dialects.

As done previously, we first conducted a comparison between dialects of those BCTs with type 1 or type 2 relation, and then we conducted a within-dialect analysis of the relative order of the BCTs compared between females and males.

Between dialect relative order comparisons showed significant differences only for *rojo* “red” (χ^2^ = 6.68, *p* < 0.05), *gris* “gray” (χ^2^ = 4.89, *p* < 0.05), *marrón/café* “brown” (χ^2^ = 6.44, *p* < 0.05), and *morado/violeta* “purple” (χ^2^ = 8.18, *p* < 0.05). Complementary Mann-Whitney *U*-tests revealed that the order of these terms was consistently higher in the Castilian sample than in either Uruguayan or Mexican samples (i.e., these terms appeared later in the Castilian sample lists). Significant differences were found in the following cases: *Rojo* “red,” Castilian vs. Uruguayan (*U* = 799.5, *p* < 0.01). *Gris* “gray,” Castilian vs. Mexican (*U* = 832, *p* < 0.05). *Marrón/Café* “brown,” Castilian vs. Mexican (*U* = 799.5, *p* < 0.01). *Morado/violeta* “purple,” Castilian vs. Mexican (*U* = 823.5; *p* < 0.01), Castilian vs. Uruguayan (*U* = 519, *p* < 0.05).

To test whether primary BCTs appeared earlier than derived BCTs, we conducted four Wilcoxon tests considering the global group and each dialect group separately. Significant differences between primary and derived BCTs were found in all cases. The means, *Z* and probability values were: Global, primary = 0.39, derived = 0.58, *Z* = −9.79, *p* < 0.001. Castilian, primary = 0.40, derived = 0.65, *Z* = −4.19, *p* < 0.001. Mexican, primary = 0.39, derived = 0.54, *Z* = −6.48, *p* < 0.001. Uruguayan, primary = 0.38, derived = 0.60, *Z* = −5.96, *p* < 0.001.

To compare the relative order of primary and the relative order of derived BCTs between females and males, Mann-Whitney *U*-tests were performed for the global group and each dialect group separately. Only three comparisons were statistically significant. In the global group, primary BCTs appeared earlier in the lists for males than for females (0.36 vs. 0.40, *U* = 172, *p* < 0.05), although this difference was only significant for the Mexican group (0.36 vs. 0.41, *U* = 172, *p* < 0.05). The female Uruguayan use of derived BCTs occurred significantly earlier in the lists than male Uruguayan use (0.58 vs. 0.66, *U* = 172, *p* < 0.05).

The results of pairwise comparisons between the relative order of BCTs for Castilian, Mexican and Uruguayan samples are detailed below.

For within-dialect comparison on the BCTs relative order we conducted a series of Wilcoxon analyses. All the chromatic primaries (*verde* “green,” *azul* “blue,” *rojo* “red,” and *amarillo* “yellow”) appeared significantly before any other BCT in the three dialects (*p* < 0.05), with two exceptions: *Amarillo* “yellow” and *morado* “purple” in the Mexican sample (*Z* = −1.62, *p* > 0.05); *Verde* “green” and *violeta* “purple” in the Uruguayan sample (*Z* = − 1.77, *p* > 0.05).

Regarding the chromatic primary BCTs, two pairs had no significant differences between in-pair members: *azul* “blue” and *rojo* “red” (pair 1); *verde* “green” and *amarillo* “yellow” (pair 2; *p* > 0.05). Pair 1 members appeared significantly earlier than pair 2 members (*p* < 0.05). The only exception to this pattern was that *verde* “green” had a similar relative order to that of *azul* “blue” and *rojo* “red” in the Castilian sample (*p* > 0.05).

Figure 3 shows that *negro* “black” and *blanco* “white,” the two achromatic primary BCTs, tended to appear in the lists earlier than derived BCTs. Wilcoxon tests indicated that such differences only were significant (*p* < 0.05) between *negro* “black” and *gris* “gray” and *marrón/café* “brown” in the three dialects. *Blanco* “white” appeared significantly earlier than these two derived categories in the Castilian, and earlier than *marrón* “brown” in the Uruguayan.

#### Discussion

Current results on the Castilian are in agreement with those previously provided by Lillo et al. (2007) and Uusküla and Bimler (2016) on the same Spanish dialect. It includes the following 11 BCTs (in italics before hyphen and their English equivalents): *verde* “green,” *azul* “blue,” *rojo* “red,” *amarillo* “yellow,” *negro* “black,” *blanco* “white,” *gris* “gray,” *naranja* “orange,” *rosa* “pink,” *marrón* “brown,” *morado* “purple”. The first 6 terms are used for naming the 6 Hering's elemental sensations and identify primary BCTs. The last terms identify derived BCTs.

Our data showed consistent differences between primary and derived BCTs. First, all the Castilian primary BCTs were also BCTs in the other two dialects, but it was not the case for the derived BCTs *marrón* “brown” and *morado* “purple” (two out of five). Second, in the three dialects, primary BCTs were more frequent than derived terms. Third, chromatic primary BCTs appeared earlier than any other BCT and achromatic primary BCTs tended to appear earlier than derived BCTs. Although results for the three dialects were broadly similar, two important differences must be highlighted. First, *celeste* “sky blue” was a BCT only for the Uruguayan where it behaved as a derived BCT both in frequency (Figure 2) and in relative order (Figure 3). Second, *blanco* “white” behaved as a derived term only in the Mexican both in frequency (Figure 2) and in relative order (Figure 3). It is also remarkable that *morado/violeta* “purple” was more frequent (Figure 2) and appeared earlier (Figure 3) in Mexican and Uruguayan than in Castilian.

There was a minor difference between our current results and those provided by Uusküla and Bimler (2016). Such difference was related to *morado* “purple” and *violeta* “purple.” In our current work, only *morado* “purple” was over fifty per cent (61.70%), which did not occur with *violeta* “purple” (40.43%). Both terms were over 50% in our previous work (Lillo et al., 2007; experiment 1; morado “purple” = 57.7%; violeta “purple” = 53.8%). Uusküla and Bimler (2016) observed that *violeta* “purple” was more frequent than *morado* “purple” in the Castilian sample. This situation is similar to that of the Uruguayan dialect, for which *violeta* “purple” (89.47%) clearly predominated over *morado* “purple” (3.51%), but not for the Mexican dialect (*morado* “purple,” 84.54%; *violeta* “purple,” 39.18%; see Table 2). In short, different studies support an important shared use of *morado* “purple” and *violeta* “purple” for Castilian and Mexican but not for Uruguayan.

### Experiment 2: extremes naming and boundary delimitation

The second experiment included two tasks: extremes naming and boundary delimitation. Both tasks were used for finding out the relationship between two BCTs retrieved from different dialects. As we commented before, there were three possible relation types between two different dialects' BCTs: (1) Equality, (2) Equivalence, (3) Difference. We considered only two possibilities for the within dialect comparisons: (1) Difference (different terms for different colors). (2) Synonymy (two different terms named the same colors).

#### Participants

Ninety students (45 females, 45 males) from three different universities (Universidad Complutense de Madrid, Spain; Universidad de Guadalajara, México; and Universidad de la República, Montevideo, Uruguay) performed both tasks included in the experiment 2. Table 3 shows their distribution by country and sex. There were no significant age differences (*p* > 0.05) between groups. Participants color vision was tested using the Ishihara color test (Ishihara, 2006) and those participants with red-green color deficient vision were excluded. None of them had protan nor deutan color vision deficiency. The research was conducted according to the principles expressed in the Declaration of Helsinki and all participants gave written informed consent. In all three universities, the research was approved by the relevant ethics committee: Hospital Clínico San Carlos Review Board in the Universidad Complutense de Madrid (Spain); Research Ethics Committee of the Faculty of Psychology in the Universidad de la República (Montevideo Uruguay); Scientific Committee of the Center of Art, Architecture and Design in the Universidad de Guadalajara (Mexico).

**Table 3:** Details of participants in Experiment 2.

**Dialect**	**Females**	**Males**	**Total**
Castilian (UCM, Madrid, Spain)	15 (20.67; 2.05)	15 (20.53; 2.83)	30 (20.60; 2.51)
Mexican (U. Guadalajara; Mexico)	15 (20.73; 2.62)	15 (20.20; 2.47)	30 (20.47; 2.59)
Uruguayan (U. de la República; Montevideo, Uruguay)	15 (20.27; 1.81)	15 (20.87; 2.00)	30 (20.95; 2.06)
Total	45 (20.47; 2.09)	45 (20.62; 2.43)	90 (20.54; 2.27)

*The rows contain the number of participants in the three samples: Castilian, Mexican or Uruguayan (mean and standard deviation of age in years in brackets)*.

#### Stimuli and apparatuses

**Figure 4:**
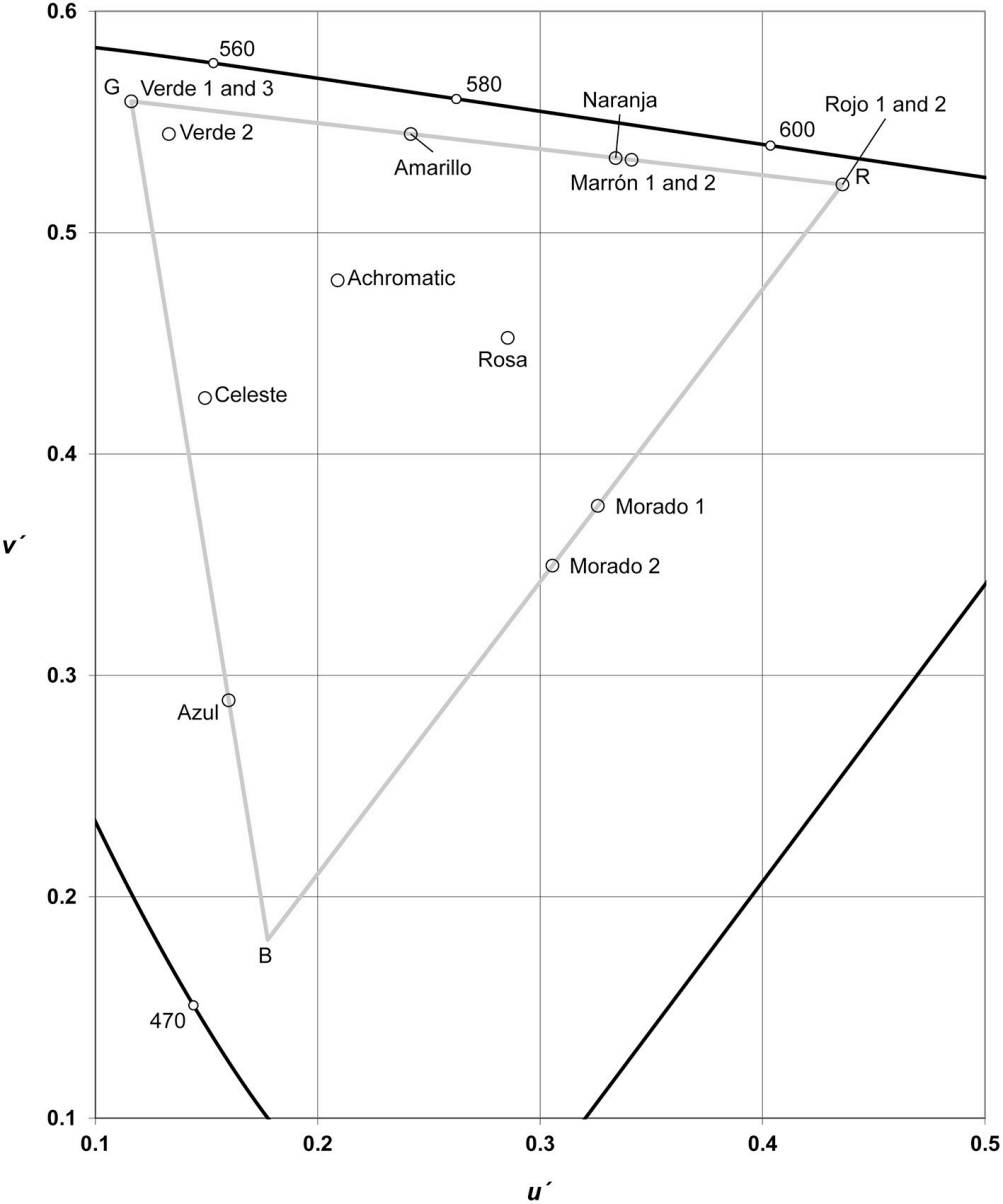
CIE *u*′*, v*′ chromatic coordinates of the 19 stimuli that were used in the extremes of the 34 color transitions (Experiment 2). Gray triangle vertices correspond to the screen primaries (red green and blue noted as R, G, and B). The achromatic point represents five stimuli (3 grays, one black, and one white).

Figure 4 shows a CIE *u*′*, v*′ chromaticity diagram (Hunt and Pointer, 2011, Chapter 3) including the chromatic coordinates of every color used in the extremes of the color transitions. The three triangle vertices (R, G, and B) correspond to the three primaries of the reference screen (red, *u*′ = 0.44, *v*′ = 0.52; green, *u*′ = 0.12, *v*′ = 0.56, blue, *u*′ = 0.18, *v*′ = 0.18) and define the “triangle of reproducible colors” (op. cit., Figure 3.14). That is, it includes the colors that can be produced by this specific screen. In our research, the indicated chromatic coordinates and the reference gamma value (2.38) resulted from the selection of very similar and accurately calibrated 21” screens (two Sony Trinitron Multiscan. One Samsung, Syncmaster). Their colorimetric calibration was performed using a Minolta CL 200 luxocolorimeter, with the accurate screen accessory, in Madrid (Spain) and Guadalajara (México). A CRS ColorCAL MKII Colorimeter was used in Montevideo (Uruguay). Each screen was used in a dim room with illuminance levels near 5 luxes. Illuminance measurements were performed with the CL 200 luxocolorimeter (Madrid and Guadalajara) and with a TES 100 luxometer (Montevideo). Observers were 50 centimeters away from the screen. It projected a visual size of 40.81° (screen diagonal). The full transition (see Figure 1) projected angles of 26.38 × 9.98°. The area limited by the whitish gray rectangle was 0.92° × 5.94°.

Table 4 contains information about the categories used in the color transition extremes. The included terms are the Castilian BCTs (Lillo et al., 2007) and *celeste* “sky blue.” Let us examine the column that corresponds to V that stands for *Verde* “Green.” As seen from Table 4, this stimulus appeared as an extreme of seven color transitions whose other extreme was one of the following categories: Az for *Azul* “Blue,” Am for *Amarillo* “Yellow,” Ne for *Negro* “Black,” B for *Blanco* “White,” G for *Gris* “Gray,” Ma for *Marrón* “Brown,” C for *Celeste* “Sky blue.” The intersection cells contain a specific symbol for the type of the stimuli. “X” indicates that both stimuli were an approximation in lightness and saturation to the Castilian best representative colors (op. cit., Table 4), except *celeste* “sky blue.” Spanish term followed by 2 or 3 indicates the stimulus whose lightness and/or chromaticity coordinates differed from the best exemplar. For example, *Verde 2* “Green *2*” in the intersection between V for *Verde* “Green” and B for *Blanco* “White” indicates that the used green had chromatic coordinates similar to those of the best exemplar of green, but different lightness (L^*^ = 86.53 instead of 31.87).

**Table 4:** Categories and stimuli used in the color transition extremes in Experiment 2.

**BCC**	**V**	**Az**	**R**	**Am**	**Ne**	**B**	**C**	**G**	**Na**	**Rs**	**Ma**	**Mo**
V												
Az	X											
R												
Am	X											
Ne	X	X										
B	*Verde*2			X								
C	*Verde*3	X				X						
G	X	X			X	X	*Gris*2					
Na			X	X								
Rs			X			X		*Gris*3	X			
Ma	X		*Rojo*2	X	X			X	X	*Brown*2		
Mo		X	*Rojo*2		X		*Morado*2	X		X	X	

The first row and the first column show the abbreviations for the Basic Color Categories (BCC): V, Verde “Green”; Az, Azul “Blue”; R, Rojo “Red”; Am, Amarillo “Yellow”; Ne, Negro “Black”; B, Blanco “White”; C, Celeste “Sky blue”; G, Gris “Gray”; Na, Naranja “Orange”; Rs, Rosa “Pink”; Ma, Marrón/Café “Brown”; Mo, Morado/Violeta “Purple.”

*The “X” symbol indicates that both stimuli were an approximation to the Castilian best representatives of each color (see lightness and chromatic coordinates in Table 5). A denomination followed by 2 or 3 indicates that the stimulus had similar coordinates and different lightness than the best representative (see Table 5)*.

Table 5 provides a complete colorimetric specification of the 19 stimuli used in the extremes of the 34 color transitions (see Figure 1). To facilitate comparisons with other publications we provide the following information: chromatic coordinates (*u*′*, v*′), hue angle value (*h*_*uv*_) computed from the *u*′*, v*′ coordinates and lightness (L^*^). Table 5, first column indicates the stimulus No. (from 1 to 19) and second column indicates the corresponding BCT. For instance, as seen from Table 5, there were three green stimuli in the color transition extremes (see the above paragraph and Table 4). The stimulus No. 1 (*Verde* “Green 1”) was the most accurate approximation provided by our screen to the best green found in our previous research (Lillo et al., 2007, Table 4). The other two greens had very similar *u*′*, v*′ coordinates (near *u*′ = 0.12 and *v*′ = 0.56) to the stimulus No. 1, but different lightness (L^*^). We used two lighter greens (stimuli No. 2 and 3) to reduce lightness changes in the transitions and, more important, the possibility of identifying more than one color category in each color transition.

**Table 5:** Colorimetric specification of the 19 colors used in the transition extremes (Experiment 2).

**No./BCT**	***u′***	***v′***	***h*_uv_**	L^*^
1 *Verde* “Green” 1	0.12	0.56	135	31.87
2 *Verde* “Green” 2	0.13	0.54	143	86.53
3 *Verde* “Green” 3	0.12	0.56	138	73.47
4 *Azul* “Blue”	0.16	0.29	225	33.34
5 *Rojo* “Red” 1	0.44	0.52	10	52.47
6 *Rojo* “Red” 2	0.44	0.52	10	41.36
7 *Amarillo* “Yellow”	0.24	0.54	64	88.72
8 *Negro* “Black”	0.21	0.48	–	0.00
9 *Blanco* “White”	0.21	0.48	–	100
10 *Celeste* “Sky blue”	0.15	0.43	220	68.13
11 *Gris* “Gray” 1	0.21	0.48	–	34.78
12 *Gris* “Gray” 2	0.21	0.48	–	71.22
13 *Gris* “Gray” 3	0.21	0.48	–	51.14
14 *Naranja* “Orange”	0.33	0.53	23	62.13
15 *Rosa* “Pink”	0.29	0.45	339	72.37
16 *Marrón*/*Café* “Brown” 1	0.34	0.53	21	21.48
17 *Marrón*/*Café* “Brown” 2	0.34	0.53	21	40.21
18 *Morado*/*Violeta* “Purple” 1	0.33	0.38	320	22.83
19 *Morado*/*Violeta* “Purple” 2	0.31	0.35	308	46.03

The first column contains the identification number of the stimulus (No.) followed by the BCT used by the participants to name each stimulus (and their English equivalent). 1 stands for the best representative, and 2 or 3 for stimuli differing in lightness. The following variables were used: CIE u′, v′ chromatic coordinates (u′, v′), CIE u′, v′ hue angle (h_uv_) and lightness (L^*^).

#### Procedure

Participants were asked to perform two tasks with each color transition. In the first task (extremes color naming task) they had to name the colors of the extremes of each transition. The instructions were to name the left extreme first and to use only one word (monolexemic naming) of common use in the everyday life (“so everybody could understand you”). After completing the extremes naming task they proceeded to the second task (boundary delimitation task). Here the instruction was to move the whitish gray rectangle (Figure 1) until place its inner part on the area of the transition they would consider the best representative color of the boundary between the two previously named categories. That is, a color that could be named using both (in combination and/or with the same probability). Such color could fall in any position between the two transition extremes. The initial whitish gray rectangle position randomly changed from trial to trial. The participants completed four training trials before moving on to the experimental trials.

#### Results

##### Task 1. Extremes naming

Table 6 shows females' (F) and males' (M) coherent naming for each stimulus used in the color transition extremes. We use the expression “coherent naming” to mean that the used term appears in Table 5 (second column). Because of naming differences between dialects, the results of stimulus 16, 17, 18, 19, and 10 are presented in two rows (each one for a different term).

**Table 6:** Female (F) and male (M) participants' coherent naming percentages for the color transition extremes.

**No**.	**Term**	**Castilian %**		**Mexican %**		**Uruguayan %**	
		**F**	**M**	**F**	**M**	**F**	**M**
1	*Verde* “Green”	100.0	100.0	100.0	100.0	100.0	100.0
2	*Verde* “Green”	100.0	100.0	100.0	100.0	100.0	100.0
3	*Verde* “Green”	100.0	100.0	100.0	100.0	100.0	100.0
4	*Azul* “Blue”	100.0	100.0	100.0	100.0	98.67	98.67
5	*Rojo* “Red”	96.67	100.0	100.0	100.0	100.0	100.0
6	*Rojo* “Red”	100.0	96.67	100.0	100.0	96.67	96.67
7	*Amarillo* “Yellow”	100.0	100.0	100.0	100.0	100.0	100.0
8	*Negro* “Black”	88.00	98.67	93.33	100.0	96.00	100.0
9	*Blanco* “White”	100.0	100.0	100.0	100.0	100.0	98.67
10	*Celeste* “Sky blue”	02.67	09.33	0	0	85.33	90.67
10	*Azul* “Blue”	97.33	88.00	100.0	100.0	09.33	05.33
11	*Gris* “Gray”	100.0	100.0	100.0	100.0	98.89	97.78
12	*Gris* “Gray”	93.33	100.0	100.0	100.0	100.0	93.33
13	*Gris* “Gray”	100.0	100.0	100.0	100.0	100.0	93.33
14	*Naranja* “Orange”	100.0	100.0	100.0	100.0	100.0	100.0
15	*Rosa* “Pink”	100.0	100.0	100.0	100.0	100.0	100.0
16	*Marrón* “Brown”	99.05	99.05	13.33	06.67	97.14	99.05
16	*Café* “Brown”	0	0	86.67	93.33	0	0
17	*Marrón* “Brown”	60.00	73.33	13.33	06.67	80.00	80.00
17	*Café* “Brown”	0	0	86.67	93.33	0	0
18	*Morado* “Purple”	73.33	82.22	60.00	66.67	12.22	12.22
18	*Violeta* “Purple”	20.00	13.33	20.00	26.67	78.89	80.00
19	*Morado* “Purple”	60.00	60.00	60.00	66.67	0	13.33
19	*Violeta* “Purple”	26.67	20.00	20.00	26.67	73.33	53.33

*The first column shows the number of the stimulus (No.). The colorimetric specification of these stimuli can be seen in Table 5. The second column contains the terms the participants labeled each stimulus with (Term). The remaining columns provide information on dialect and sex distribution of the participants making each stimulus-term pair*.

To understand the data presented in Table 6 it is important to keep in mind that the same percent can correspond to different absolute frequencies. For example, the stimulus No. 1 (*Verde* “Green 1”; see Table 5) was an extreme in 5 transitions, and every participant always named this stimulus *verde* “green.” This stimulus scored 100% of coherent naming with an absolute frequency equal to 75 (all the women, 15, used *verde* “green” in five color transitions). The stimulus No. 2 (*Verde* “Green 2”; see Table 5) also scored 100% though its absolute frequency was equal to 15 (it was an extreme in only one ransition).

We conducted χ^2^ analyses to test whether the extremes naming was dependent on sex or dialect. Female vs. male comparisons only showed significant differences for stimuli No. 8 (*negro* “black,” χ^2^ = 15.39, *p* < 0.05) and 18 (*morado/violeta* “purple” 1, χ^2^ = 21.98, *p* < 0.01). Pairwise comparison only showed significantly greater frequencies for men than for women in the Mexican sample (*negro* “black,” χ^2^ = 5.17, *p* < 0.05; *morado/violeta* “purple” 1, χ^2^ = 13.17, *p* < 0.01).

Comparisons between dialects only showed significant differences in the naming of 5 stimuli; No. 10 (*celeste/azul* “sky blue,” χ^2^ = 359.53, *p* < 0.001), 16 (*marrón/café* “brown,” χ^2^ = 549.08, *p* < 0.001), 17 (*marrón/café* “brown,” χ^2^ = 86.60, *p* < 0.001), 18 (*morado/violeta* “purple,” χ^2^ = 247.21, *p* < 0.001), and 19 (*morado/violeta* “purple,” χ^2^ = 33.44, *p* < 0.001). Pairwise comparisons showed that there were no significant differences (*p* > 0.05) between the Castilian and the Mexican in the naming of stimulus 10, but they differed (*p* < 0.05) from the Uruguayan. In the latter case, the use of *celeste* “sky blue” predominated over *azul* “blue,” but *azul* “blue” predominated (Castilian) or was the only used term (Mexican) in the other two dialects. No significant differences were found (*p* > 0.05) between Castilian and Uruguayan for stimuli 16 and 17 (named *marrón* “brown” and *café* “brown”), but both dialects differed from Mexican (*p* < 0.05). *Café* “brown” was used more frequently in Mexican and *marrón* “brown” was used more in Castilian and Uruguayan. For stimuli 18 and 19 there were no differences (*p* > 0.05) in the use of *morado* “purple” and *violeta* “purple” between Castilian and Mexican, but both differed significantly (*p* < 0.05) from Uruguayan. For Uruguayan the predominant term was *violeta* “purple,” but for Castilian and Mexican it was *morado* “purple”.

##### Task 2. Boundary delimitation task

**Figure 5:**
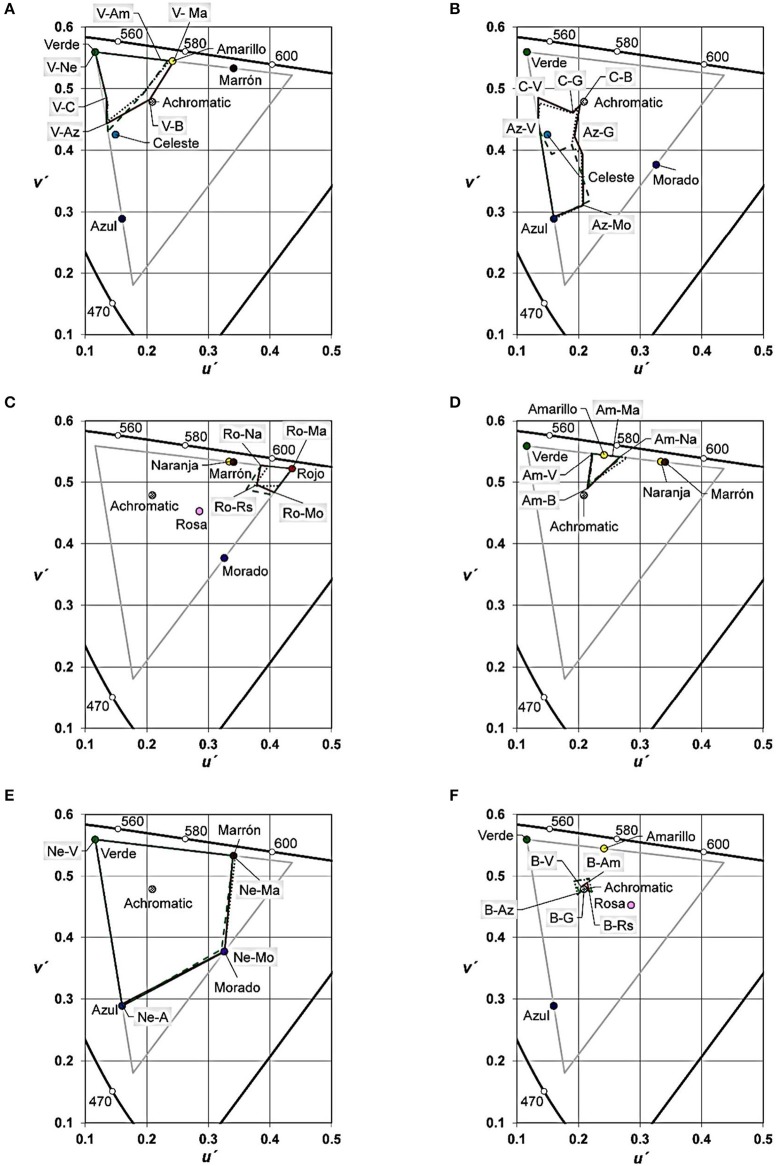
CIE *u*′*, v*′ colorimetric delimitation of the primary BCTs for the three dialects in Experiment 2. **(A)**. *Verde* “Green.” **(B)** *Azul* “Blue.” **(C)** *Rojo* “Red.” **(D)** *Amarillo* “Yellow.” **(E)** *Negro* “Black.” **(F)** *Blanco* “White.” Colored circles represent the stimuli of the extremes of color transitions. The achromatic point shows the coordinates of the best exemplars of *negro* “black,” *blanco* “white” and *gris* “gray.” Each diagram included 3 lines (one per dialect: Castilian, solid line; Mexican, dotted line; Uruguayan, dashed line) obtained after connecting the boundaries of the target category using minimum angular direction changes. They delimitate the chromatic surface of each BCC in each dialect. Labels with gray background indicate boundary locations (V stands for *Verde* “Green”; Az for *Azul* “Blue”; R for *Rojo* “Red”; Am for *Amarillo* “Yellow”; Ne for *Negro* “Black”; B for *Blanco* “White”; C for *Celeste* “Sky blue”; G for *Gris* “Gray”; Na for *Naranja* “Orange”; Rs for *Rosa* “Pink”; Ma for *Marrón/Café* “Brown”; Mo for *Morado/Violeta* “Purple”). Gray triangle vertices correspond to the screen primaries.

Figures 5 (primary BCTs), **6** (derived BCTs) provide a colorimetric delimitation for the 12 BCCs of the Spanish language. Each diagram identifies the stimuli used in the color transitions belonging to the target BCC and the neighbor BCCs. The achromatic point shows the coordinates of the best examples of *negro* “black,” *blanco* “white” and *gris* “gray.” Each diagram presents a line per dialect (Castilian, solid line; Mexican, dotted line; Uruguayan, dashed line). These lines were determined by connecting the boundaries between the target category and its neighbors using the minimum angular changes from point to point. We name such lines “chromatic contours,” and they delimit the chromatic surfaces for each BCC.

**Figure 6:**
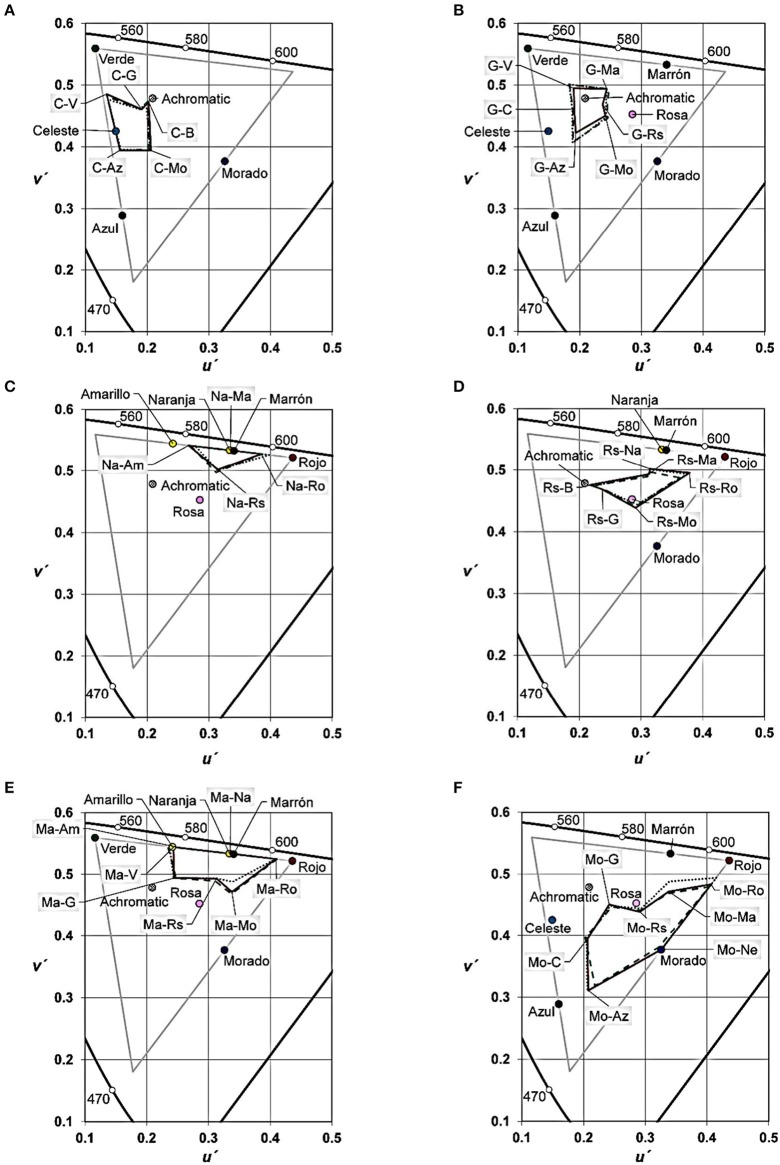
CIE *u*′, *v*′ colorimetric delimitation of the derived BCTs for the three dialects in Experiment 2. **(A)** *Celeste* “Sky blue.” **(B)** *Gris* “Gray.” **(C)** *Naranja* “Orange.” **(D)** *Rosa* “Pink.” **(E)** *Marrón/Café* “Brown.” **(F)** *Morado/Violeta* “Purple.” Colored circles represent the stimuli of the extremes of color transitions. The achromatic point shows the coordinates of the best exemplars of *negro* “black,” *blanco* “white” and *gris* “gray.” Each diagram included 3 lines (one per dialect: Castilian, solid line; Mexican, dotted line; Uruguayan, dashed line) obtained after connecting the boundaries of the target category using minimum angular direction changes. They delimitate the chromatic surface of each BCC in each dialect. Labels with gray background indicate boundary locations (V stands for *Verde* “Green”; Az for *Azul* “Blue”; R for *Rojo* “Red”; Am for *Amarillo* “Yellow”; Ne for *Negro* “Black”; B for *Blanco* “White”; C for *Celeste* “Sky blue”; G for *Gris* “Gray”; Na for *Naranja* “Orange”; Rs for *Rosa* “Pink”; Ma for *Marrón/Café* “Brown”; Mo for *Morado/Violeta* “Purple”). Gray triangle vertices correspond to the screen primaries.

We will use the Figure 5A diagram to explain how the information is provided by Figures 5, 6. Figure 5A (*Verde* “Green”) shows the chromatic coordinates of the three green stimuli we used in the transition extremes (“Verde”) and the color coordinates of the other extreme of the same transitions (“Azul,” “Achromatic,” “Amarillo,” etc. see Table 4). The chromatic contour vertices are the mean localization of the green boundaries, and the adjacent abbreviation identifies the bordering BCCs, e.g., V-Am (*Verde*-*Amarillo*, i.e., Green-Yellow), V-Ma (*Verde*-*Marrón*, i.e., Green-Brown), etc.

Figures 5, 6 do not differentiate between females and males. We conducted a series of Mann-Whitney *U*-tests for comparing the chromatic coordinates (*u*′*, v*′ values) of the 34 color transitions in each dialect (136 comparisons = 34 transitions × 2 variables × 2 analysis types). We only found 16 significant differences (*p* < 0.05) between males and females: 2 in the Castilian sample, 6 in the Mexican sample, and 8 in the Uruguayan sample. Even for these cases the differences were very small (Δ*u*′ or Δ*v*′ < 0.02), so we decided not to differentiate between females and males in the following analysis with *u*′ and *v*′.

The chromatic areas (Figures 5, 6) were very similar across the three dialects, except for *Azul* “Blue” (Figure 5B). Such similarity was confirmed by the results of 72 non parametric Kruskal-Wallis variance analyses followed, when accurate, by Mann-Whitney *U*-tests to conduct pairwise comparisons between dialects. Table 7 left column resumes the results of the 16 out of 34 color transitions where at least one significant difference (*p* < 0.05) appeared. Only 43 out of all the possible comparisons were significant (21.43%).

**Table 7:** Significant differences between the three dialects (Castilian, Mexican, Uruguayan) in the *u*′, *v*′ values of the boundary colors provided by the Experiment 2.

	**Castilian**		**Mexican**		**Uruguayan**		**Castilian Mexican**		**Castilian Uruguayan**		**Mexican Uruguayan**	
**Transition**	***u*′**	***v*′**	***u*′**	***v*′**	***u*′**	***v*′**	**Δ*u*′**	**Δ*v*′**	**Δ*u*′**	**Δ*v*′**	**Δ*u*′**	**Δ*v*′**
V-Az	0.13	0.44	0.13	0.45	0.14	0.43	0.00	0.00	0.00^*^	0.01^*^	0.00^*^	0.01^*^
V-B	0.20	0.48	0.19	0.49	0.19	0.49	0.011^*^	0.010^*^	0.011^*^	0.009	0.00	0.00
V-G	0.19	0.49	0.18	0.50	0.18	0.50	0.007^*^	0.006^*^	0.007^*^	0.01^*^	0.00^*^	0.00
V-C	0.13	0.49	0.14	0.48	0.14	0.48	0.002^*^	0.009	0.00	0.00	0.00	0.00
Az-Mo	0.21	0.31	0.21	0.31	0.22	0.32	0.00	0.00	0.01	0.01^*^	0.012	0.008^*^
Az-G	0.19	0.42	0.19	0.41	0.19	0.41	0.005^*^	0.017^*^	0.004^*^	0.01	0.00^*^	0.00
R-Na	0.39	0.53	0.40	0.53	0.39	0.53	0.012^*^	0.001^*^	0.00	0.00	0.012^*^	0.001^*^
R-Rs	0.38	0.50	0.37	0.49	0.36	0.49	0.00	0.00	0.017^*^	0.01^*^	0.013^*^	0.006^*^
R-Mo	0.41	0.48	0.41	0.49	0.40	0.48	0.008^*^	0.010^*^	0.00	0.00	0.011^*^	0.014^*^
Am-Na	0.27	0.54	0.28	0.54	0.27	0.54	0.011^*^	0.001^*^	0.00	0.00	0.008^*^	0.001^*^
Am-B	0.26	0.54	0.26	0.54	0.26	0.54	0.00^*^	0.00^*^	0.00^*^	0.00^*^	0.00	0.00
C-B	0.20	0.47	0.20	0.47	0.20	0.47	0.004^*^	0.00^*^	0.00	0.00	0.00^*^	0.00
Na-Rs	0.26	0.54	0.26	0.54	0.26	0.54	0.00	0.00	0.00^*^	0.00^*^	0.00^*^	0.00^*^
Rs-B	0.22	0.48	0.22	0.47	0.22	0.47	0.006^*^	0.002^*^	0.00	0.00	0.00	0.00
Rs-G	0.24	0.47	0.24	0.47	0.24	0.47	0.01	0.00	0.008^*^	0.01^*^	0.00	0.00
Mo-Ma	0.34	0.47	0.34	0.49	0.34	0.47	0.00^*^	0.016^*^	0.00	0.00	0.001^*^	0.019^*^

* *p < 0.05*.

The columns under “Castilian,” “Mexican,” and “Uruguayan” show the mean u′ and v′ settings for each dialect in the u′, v′ boundaries values. The columns under “Castilian-Mexican,” “Castilian-Uruguayan,” and “Mexican-Uruguayan” show the mean difference (Δ) between the u′ and v′ settings for the named pair of dialects. An asterisk indicates a significant difference between the settings according to a Mann-Whitney U-test. V, Verde “Green”; Az, Azul “Blue”; R, Rojo “Red”; Am, Amarillo “Yellow”; Ne, Negro “Black”; B, Blanco “White”; C, Celeste “Sky blue”; G, Gris “Gray”; Na, Naranja “Orange”; Rs, Rosa “Pink”; Ma, Marrón/Café “Brown”; Mo, Morado/Violeta “Purple.”

Figure 5B is the only diagram with important differences in the shape and the extension of the chromatic areas. As seen from this figure, the areas of the Castilian *Azul* “Blue” and the Mexican *Azul* “Blue” are similar, but it is not the case of the Uruguayan *Azul* “Blue.” The information provided by Figure 6A (*Celeste* “Sky blue”) is related to the aforementioned results.

Though there are three chromatic contours in Figure 6A (one per dialect), only the dashed line (Uruguayan) corresponds to a real BCT-BCC. That is, each vertex of this chromatic contour results from the mean *u*′, *v*′ values of a color transition where one extreme was consistently named celeste. The speakers of the other two dialects named this color *azul* “blue.” Moreover, in the specific case of the boundary labeled “C-Az” most of the Castilian and Mexican participants indicated that, this time, the transition included two instances of the same blue category, so we invited them to consider the colors as good exemplars of two different categories, and to complete the task.

**Figure 7:**
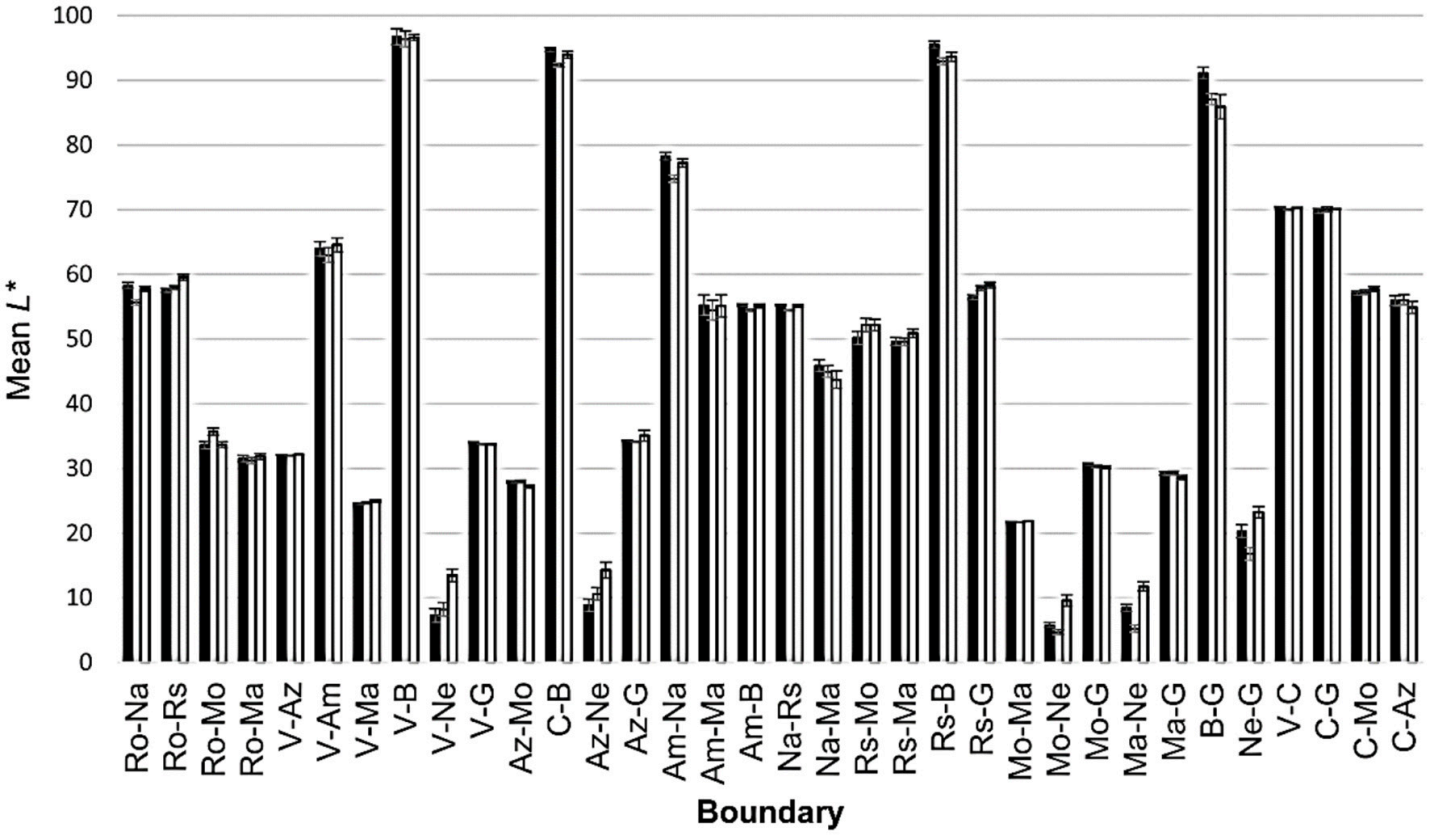
Mean L^*^ values (*y*-axis), adjusted for the boundaries (*x*-axis), identified in each color transition in Experiment 2. V stands for *Verde* “Green”; Az for *Azul* “Blue”; R for *Rojo* “Red”; Am for *Amarillo* “Yellow”; Ne for *Negro* “Black”; B for *Blanco* “White”; C for *Celeste* “Sky blue”; G for *Gris* “Gray”; Na for *Naranja* “Orange”; Rs for *Rosa* “Pink”; Ma for *Marrón/Café* “Brown”; Mo for *Morado/Violeta* “Purple.” Error bars show the standard error of the mean (± SEM).

Figure 7 bars provide mean L^*^ values for the identified boundaries in each color transition. The bars are chunked in groups of three (Castilian left, Mexican center, Uruguayan right). To facilitate comparisons between dialects, Table 8 not only informs about the specific L^*^ values of each transition, but it also shows the differences in L^*^ values (ΔL^*^) between dialects. Such differences were of much reduced magnitude. We conducted a series of Mann-Whitney *U*-tests finding that 41.17% (14/34) of the comparisons between Castilian and Mexican were significant, 32.35% (11/34) of the comparisons between Castilian and Uruguayan were significant, and 35.29% (12/34) of the comparisons between Mexican and Uruguayan were significant. The biggest ΔL^*^ values were 4.06 in the Castilian-Mexican comparisons, 6.17 in the Castilian-Uruguayan comparisons and 6.49 in the Mexican-Uruguayan comparisons.

**Table 8:** Significant differences between the three dialects (Castilian, Mexican, Uruguayan) in the L^*^ values provided by the Experiment 2.

	**Castilian**	**Mexican**	**Uruguayan**	**Castilian Mexican**	**Castilian Uruguayan**	**Mexican Uruguayan**
**Transition**	L^*^	L^*^	L^*^	ΔL^*^	ΔL^*^	ΔL^*^
V-B	96.75	96.38	96.61	0.36^*^	0.14	0.23
V-Ne	07.28	08.22	13.45	0.94	6.17^*^	5.23^*^
V-G	34.05	33.74	33.73	0.31^*^	0.32^*^	0.00
V-Az/C	70.42	70.02	70.28	0.40^*^	0.14	0.26^*^
Az-Mo	27.91	27.99	27.16	0.08	0.74^*^	0.83^*^
Az/C-B	94.78	92.38	93.96	2.39^*^	0.82	1.58
Az-Ne	8.82	10.59	14.27	1.77	5.45^*^	3.68
Az-G	34.30	34.12	35.05	0.19^*^	0.75	0.94^*^
R-Na	58.31	55.68	57.75	2.63^*^	0.56	2.08^*^
R-Rs	57.55	57.97	59.56	0.42	2.00^*^	1.59^*^
R-Mo	33.59	35.69	33.64	2.10^*^	0.05	2.05^*^
Am-Na	78.27	74.81	77.23	3.47^*^	1.05	2.42^*^
Am-Ma	55.25	54.46	55.11	0.79^*^	0.14	0.65
Am-B	55.25	54.46	55.11	0.79^*^	0.14^*^	0.65
Ne-G	20.30	16.79	23.23	3.50	2.93	6.44^*^
B-G	91.15	87.10	85.93	4.06^*^	5.23	1.17
Na-Rs	55.25	54.46	55.11	0.79	0.14^*^	0.65
Rs-B	95.55	92.93	93.64	2.61^*^	1.90^*^	0.71
Rs-G	56.49	57.88	58.32	1.39	1.84^*^	0.44
Ma-Ne	8.43	5.25	11.75	3.18^*^	3.32^*^	6.50^*^
Mo-Ma	21.77	21.70	21.84	0.07	0.07	0.14^*^
Mo-Ne	5.79	4.66	9.58	1.13	3.78^*^	4.91^*^

* *p < 0.05*.

The columns under “Castilian,” “Mexican” and “Uruguayan” show the mean L^*^ settings for each dialect. The columns under “Castilian-Mexican,” “Castilian-Uruguayan” and “Mexican-Uruguayan” show the mean difference (ΔL^*^) between the L^*^ settings for the named pair of dialects. An asterisk indicates a significant difference between the settings according to a Mann-Whitney U-test. V, Verde “Green”; Az, Azul “Blue”; R, Rojo “Red”; Am, Amarillo “Yellow”; Ne, Negro “Black”; B, Blanco “White”; C, Celeste “Sky blue”; G, Gris “Gray”; Na, Naranja “Orange”; Rs, Rosa “Pink”; Ma, Marrón/Café “Brown”; Mo, Morado/Violeta “Purple.”

## General discussion

The frequency of use of color terms in the Elicited lists (Figure 1) revealed 11 BCTs in Castilian and Mexican and 12 in Uruguayan. Therefore, unlike Japanese BCTs-BCCs (Kuriki et al., 2017), Castilian BCCs-BCTs did not evolve since our previous work (Lillo et al., 2007). This conclusion is also based upon colorimetric similarity between chromatic areas (u′, v′ values in Figures 5, 6) and lightness ranges (L^*^ values in Figure 7) that correspond to the 11 Castilian BCTs, and the results we obtained for the same variables in our previous work (op. cit., Figures 3–6). As previously found (op. cit.), our current work did not show significant differences between females and males. This is in agreement with the data from other works on BCTs (Al-Rasheed et al., 2011; Lindsey and Brown, 2014), despite the case that females tend to use more color terms than males (MacDonald and Mylonas, 2014).

Our current data show that the set of BCCs of each dialect breaks down the color space in a number of similar ways. In all three dialects, we found characteristics that define several “colorimetric signs of identity” of each BCC. For example, there is a gap between the chromatic area of red and the achromatic point (Figure 5C; compare to Lillo et al., 2007, Figure 6) and between the chromatic area of orange and the achromatic point (Figure 6C; compare to Lillo et al., 2007, Figure 5). This means that these chromatic BCCs do not include low saturated colors, and, therefore, the use of color transitions (Figure 1) enabled us to affirm that some chromatic BCCs (red and orange) do not share boundaries with the achromatic BCCs. This fact cannot be detected using the WCS color set (f.i. Kay et al., 2009; Lindsey and Brown, 2014; Brown et al., 2016; Kuriki et al., 2017) because this set includes few low saturated colors, especially for medium and medium-low lightness levels.

The size of the lightness range is another colorimetric “sign of identity.” As seen in Figure 7 and Table 8, some categories, like red, have a greatly reduced L^*^ range (between about 60 for the red-pink boundary and about 30 for the red-brown boundary). Other categories are compatible with wider L^*^ ranges. In the specific case of green, L^*^ values extended from about 95 (green-white boundary) to about 10 (green-black boundary). A third “sign of identity” is the size of the chromatic area. As in our previous work (Lillo et al., 2007, Figure 4), the chromatic area is inversely related to the lightness of the achromatic categories, i.e., the smallest area is that of white (Figure 5F), gray's area is larger (Figure 6B), and black's area is the largest one (Figure 5E). Artifacts of a limited hue resolution of the color monitors and their lower luminance ranges could be partly responsible for such large area of black. In our next work, this problem could be addressed by increasing the luminance of the black reference stimulus.

The UE model (Kay and McDaniel, 1978; Kay and Maffi, 1999; Kay et al., 2009) differentiates between primary and derived BCCs according to their presumed relationship with Hering's elemental sensations. In the UE model, primary BCCs hold a special status because they are related to the predominance of only one elemental sensation. Our results support such a special status because primary BCTs (1) were identical in all three dialects as shown in both Elicited lists (Figure 2) and extremes naming task (Table 2), and (2) tended to be more frequent (Figure 2) and appeared earlier than derived BCTs (Figure 3) in the Elicited lists. Let us comment briefly on these two latter conclusions.

The analysis of the relative positions (Figure 3) confirmed that the primary BCTs, and especially the chromatic ones, tended to appear earlier in the Elicited lists (this result is in agreement with Lillo et al., 2007, Figure 1). The analyses described in an in-press work (Lillo et al., 2018, Figure 2) show that this conclusion is also valid when absolute positions are compared (“mean position” in the nomenclature of Corbett and Davies, 1997).

Six primary BCTs and three derived BCTs were identical in all three dialects. Apart from blue, they also occupied similar volume in the color space (areas and L^*^). Using the nomenclature set out in the Introduction, the relation type was 1 (equality). On the other hand, different terms used for brown and purple revealed dialectal variations to denote these two derived BCCs, yielding a type 2 relation: equivalence. The smaller color area of blue in Uruguayan (area: Figure 5A, L^*^: Figure 7) was due to the excision of the sky blue category (dashed line in Figure 6A), which led it to be classified as a type 3 relation: difference.

The differences in volume of the blue category (Figures 5B, 7) and the presence of sky blue category in only Uruguayan (Figure 6A) are the most important inter-dialect differences. In line with LRH postulates, this substantiates the importance of linguistic-cultural factors in the evolution of languages. These differences were compared to the results from color transitions involving stimulus No. 10 as seen in Table 6, which was predominantly denoted as *azul* “blue” by Spaniards and Mexicans, but *celeste* “sky blue” by Uruguayans. The boundaries obtained from these color transitions delimited the chromatic areas of Castilian and Mexican blues, but not the Uruguayan one. This is the cause of the smaller chromatic area of Uruguayan blue in Figure 5B.

The boundaries relating to stimulus No. 10 were similar across all three dialects, and therefore defined similar volumes for the real (Uruguayan) and the hypothetical (Castilian and Mexican) sky blue (Figures 6A, 7). This indicates that the creation of this category in Uruguayan was based upon a chromatic variation that is similarly perceived by the speakers of all three dialects. This fact is in agreement with the predictions of UE and, as we it will show, the interpoint distance model (IDM, Jameson and D'Andrade, 1997).

In the IDM, the best exemplars of primary categories (white, black, red, yellow, and green) hold a special status due to their location in the color spaces defined by surface colors (CSSCs, see, Hunt and Pointer, 2011, Chapter 8), and not due to their association to Hering's elemental sensations. The colors included in CSSCs define well-known color order systems such as Munsell, NCS, and OSA (Optical Society of America). All tiles that comprise the surface color samples included in color books corresponding to these systems can be described according to CIE colorimetric variables. This enables the comparison of results between works on BCCs carried out with samples from Munsell (f.i. Sturges and Whitfield, 1995, on American British; Lindsey and Brown, 2014, on British English), NCS (f.i. Lillo et al., 2007), and OSA (f.i. Boynton and Olson, 1987). The description in terms of CIE variables also makes it possible to delimit the subset of a color space that can be reproduced with a specific screen (Figure 4; Hunt and Pointer, 2011, Figure 3.14), and, therefore, to compare data obtained from screens with surface colors.

IDM (Jameson and D'Andrade, 1997) indicates accurately that the best exemplars of BCCs tend to be located in furthest protrusions in the external surface of CSSCs. This maximizes the distance between the best exemplars of BCCs and leads to predict a sequence of apparition of BCCs similar to that predicted in UE model (op. cit., Figure 14.5). Considering the case of a possible partition of Castilian and Mexican blues, IDM correctly predicts that the best exemplars of a new category must be light (such as Uruguayan sky blue, where the best exemplars of the original blue are dark).

Figure 4 shows that the majority of the best representatives of most BCCs are located in the triangle of reproducible colors, and correspond to the maximum saturation (*s*_*uv*_; see Hunt and Pointer, 2011, Chapter 3) levels provided by our reference screen for each hue angle (*h*_*uv*_) value. Only three good chromatic representatives were inside the triangle: “Verde 2” (green), “Rosa” (pink), and “Celeste” (sky blue). These three were all light stimuli (their L^*^ values were 86.53, 72.37, and 68.13, respectively; Table 5), and their saturation level was the maximum possible value for their specific hue angle and lightness combinations.

What kind of perceptual change appears in the transition between *celeste* “sky blue” (stimulus No. 10) and *azul* “blue” (stimulus No. 4)? We think it is related to the relative proportions between the Hering's elemental sensations of blue and white. Let us assume that the *celeste* “sky blue” extreme is characterized by a perfect equilibrium between the blue and the white sensations, and that the movement toward the other transition extreme, *azul* “blue,” progressively increases the blue sensation and reduces the white one (i.e., *celeste* “sky blue” progressively becomes more bluish and less whitish). The *celeste* “sky blue” boundary would be at the point where the blue sensation becomes predominant over white. This critical point would be perceived similarly by the speakers of all three dialects (universal similarity factor), but only used as the limit of sky blue BCC by Uruguayans, because of the influence of specific linguistic-cultural factors such as considered by the LRH.

The BCT for brown in Castilian and Uruguayan dialects is *marrón*, whereas it is café in Mexican (see Tables 2, 6). The latter is in accord with Harkness's (1973) finding of *café* “brown” term in the Spanish dialect in Guatemala, bordering Mexico, with both Central American countries being at a significant distance from South American Uruguay. This variation demonstrates basic color terms originating in reference to colors of significant objects in the environment and gradually evolving into abstract concepts. Both Mexico and Guatemala are prominent coffee-growing regions, and their term to denote brown and coffee coincides: *café*. Nevertheless, regardless of the origins of the term, it remains that the color volumes of the brown category are very similar across all three dialects (Figures 6E, 7).

Considering the case of the purple category from the LRH perspective, we could postulate that the Castilian and Mexican term *morado* “purple” (Tables 1, 4) derives from the Spanish term for black mulberry (*mora*) whose plant is widely spread on Iberian Peninsula. In Uruguayan, purple is almost exclusively named *violeta* “purple” (see Tables 1, 4). Once again, regardless of the origin of the term, the color volumes of purple are similar across the three dialects (Figures 6F, 7).

*Celeste* “sky blue” was only a BCT in Uruguayan. Therefore, this dialect presented a set of 12 BCCs-BCTs. The frequency and mean position of *celeste* “sky blue” indicated that it behaved as a derived BCT (Figures 1, 2). Similarly to the results of our current work on Uruguayan, two blues, termed *azul* “blue” and *celeste* “sky blue,” were also found in works on other Rioplatense Spanish dialects spoken in Argentina, Chile, Paraguay (González-Perilli et al., 2017), and Guatemala (Harkness, 1973, Figure 9).

Our colorimetric specification for *celeste* “sky blue” was fully compatible with the data found for *azzurro* “sky blue” in Italian (Paggetti et al., 2016; Bimler and Uusküla, 2017). Of the two Italian blues, *azzurro* “sky blue” includes a subset of blues with smaller *h*_*uv*_ values and higher L^*^ values, and with a color space similar to that of Uruguayan sky blue. This similarity suggests the emergence of this second blue, termed *celeste* “sky blue,” in Uruguayan as a result of substantial Italian immigration to Uruguay during the Nineteenth and twentieth centuries (Goebel, 2010), as well as to other the South American countries (as with aforementioned Spanish dialects). Along with language contact, a linguistic factor could have played a role. The compound term “*azul celeste*” (literally, sky blue) is common in Castilian Spanish and phonologically similar to Italian term *celeste* (sky). This similarity could have driven the emergence of the new category in the Spanish dialects in question. Both language contact and linguistic factors behind the apparition of two blues in Uruguayan provide evidence in favor of the LRH.

In conclusion, our research revealed important similarities between the three Spanish dialects both in number and in colorimetric delimitation of the BCTs-BCCs. The main difference was the inclusion of *celeste* “sky blue” as a BCT-BCC in only the Uruguayan dialect. A differentiation process that segregated the light and not very saturated blues from the other colors included in a previous, more ample blue category (similar to Castilian and Mexican blue) might produce this BCT-BCC. Such a process was probably fueled by the influence of the language spoken by the Italian migrants that traveled to Uruguay in the last two centuries. The presence of *celeste* “sky blue” in Uruguayan, but not in Castilian and Mexican, makes the relevance of specific linguistic-cultural factors evident. However, the similarity between the colorimetric delimitation of the real Uruguayan sky blue category (Figure 6A, dashed line) and the hypothetical Castilian (solid line) and Mexican (dotted line) sky blue categories indicated such cultural factor do not operate on a *tabula rasa* but rather on a color space with a universal structure. In brief, our data support perceptual universalism modulated by some linguistic-cultural factors. This theoretical position could be termed “weak linguistic relativity.”

## Author contributions

JL, FG-P, and LP-L designed the study. HM and LÁ performed the stimulus creation. Data collection was performed by JL, FG-P, LP-L, and LÁ. JC, HM and AM analyzed the data. JL, HM and LÁ drafted the manuscript. All authors approved the final version of the manuscript for submission.

### Conflict of interest statement

The authors declare that the research was conducted in the absence of any commercial or financial relationships that could be construed as a potential conflict of interest.
